# Electron configuration-based neural network model to predict physicochemical properties of inorganic compounds†

**DOI:** 10.1039/d0ra05873d

**Published:** 2020-09-08

**Authors:** Hyun Kil Shin

**Affiliations:** Toxicoinformatics Group, Department of Predictive Toxicology, Korea Institute of Toxicology Daejeon 34114 Republic of Korea hyunkil.shin@kitox.re.kr +82 42 610 8276

## Abstract

Registration, evaluation, and authorization of chemicals (REACH), the regulation of chemicals in use, imposes the characterization and report of the physicochemical properties of compounds. To cope with the financial burden of the experiments, the use of computational models is permitted for prediction of properties. Although a number of physicochemical property prediction models have been developed, their applicability domain is limited to organic molecules since most available data are concerned with organic molecules, and most of the molecular descriptors are restricted to organic molecule calculations. Prediction models developed for inorganic compounds were intended to predict endpoints relevant to novel material design. Therefore, no models were available for predicting endpoints of inorganic compounds that are significant to regulatory perspectives. In this study, boiling point, water solubility, melting point, and pyrolysis point prediction models were developed for inorganic compounds based on their composition. The electron configuration of each element in the molecule was used as a descriptor in this study. The dataset covered a wide range of endpoints and diverse elements in their structure. The performance of the models was measured using *R*^2^, mean absolute error, and Spearman's correlation coefficient, and indicated good prediction accuracy of continuous endpoints and prioritization of inorganic compounds.

## Abstract 2

Registration, evaluation, and authorization of chemicals (REACH), the regulation of chemicals in use, imposes the characterization and report of the physicochemical properties of compounds.

## Graphical abstract

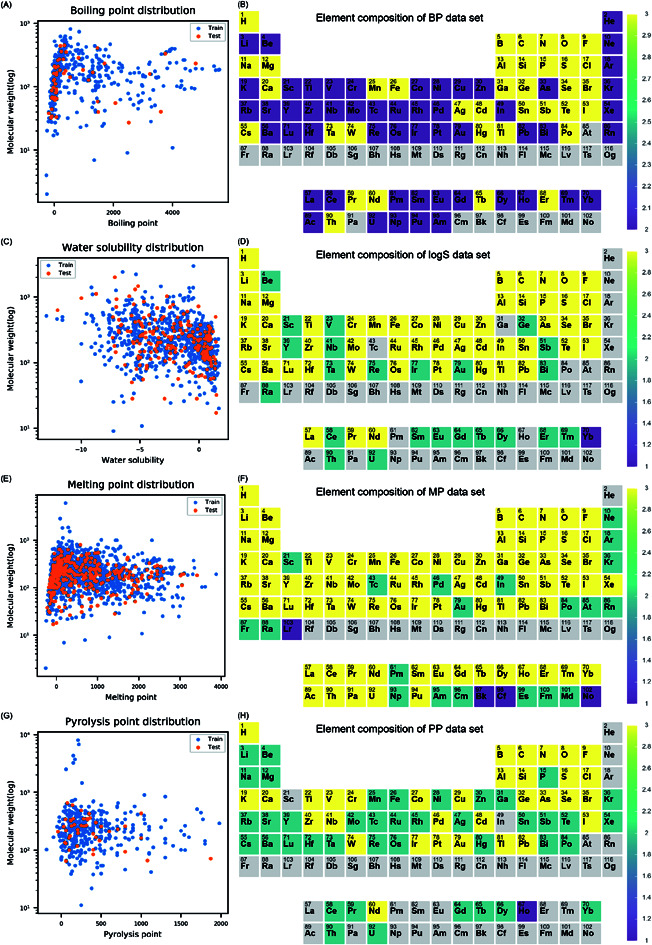

## 1. Introduction

Understanding of risks imposed by chemicals leads to the preparation of national legislation and regulations to protect environmental and human health.^1^ In Europe, registration, evaluation, and authorization of chemicals (REACH) has entered into force to manage the risk of chemicals manufactured, imported, or used. In South Korea, the act on registration and evaluation of chemicals (K-REACH) has come into effect since 2015.^2^ In the registration process, (eco)toxicological and physicochemical properties of chemicals are to be reported if they are manufactured, imported or used over one ton per year.^3,4^ Since experimental testing to measure physicochemical properties of chemicals is time consuming and high cost, it is allowed to use quantitative structure–activity/property relationship (QSAR/QSPR) models in their measurements.^5^ QSAR/QSPR models are expected to significantly decrease redundant experimental testing and to make the most out of data by exploiting accumulated chemical data.

Physicochemical properties such as normal melting point (MP), normal boiling point (BP), water solubility (log *S*), and pyrolysis point (PP) are strongly related to the behavior of chemicals in humans and the environment.^6^ These properties are to be reported for registration according to guidelines in REACH.^7^ Due to their importance, some QSPR models to predict MP, BP, log *S*, and PP have been developed. Few review articles were dedicated to evaluate available data, models, and software for the properties.^5,6,8^ However, the currently developed QSPR models predict only the properties of organic compounds. Therefore, no models are available for the prediction of properties for inorganic compounds.

Studies on prediction model development targeting inorganic molecules are increasing recently in the field of novel materials design as more efficient methodologies are required to explore the vast chemical space of inorganic compounds rapidly.^9,10^ Models developed for inorganic compounds were intended to aid novel material design aiming prediction of endpoints such as lattice thermal conductivity,^11^ electronic structure properties (quantum-mechanically-derived properties),^12–14^ crystallographic parameters,^15,16^ or material synthesis parameters.^17,18^ If prediction models for nanomaterials are excluded from the scope of discussion, no models for inorganic compounds were developed to predict endpoints significant in a regulatory perspective except one which predicts inorganic toxicity of substances toward rats.^19^

The major reason that prediction models for physicochemical properties of inorganic compounds have not been developed, is mainly related to the lack of available data. In publicly available physicochemical property datasets, the majority of data are property measurements of organic compounds, whereas inorganic compounds were rare or absent among these datasets.^20,21^ The second reason is related to the available tool for descriptor calculation. In QSAR/QSPR model development studies, a number of tools are developed to calculate descriptor or fingerprint of organic molecules,^22,23^ and these are not applicable to inorganic molecules.^20^ Novel descriptors have been developed for metal/metal oxide nanoparticles; however, these are not applicable to bulk inorganic compounds since the descriptors are calculated in a size-dependent manner. In my best knowledge, only two tools were developed for descriptor calculation of inorganic compound: Magpie (Materials-Agnostic Platform for Informatics and Exploration),^24^ and matminer.^25^ Further descriptors for inorganic compounds were prepared from fundamental information such as atom frequency^15^ or elemental properties (*e.g.*, atomic radius, electronegativity, group number, and so forth).^11^ Given that the molecular structure of inorganic compounds is more complex through a combination of diverse atoms than organic compounds, more studies are needed for the development of inorganic compound descriptor easily and rapidly applicable in machine learning model building.

In this study, neural network models were developed to predict MP, BP, log *S*, and PP based on the composition of inorganic molecules. The models cover a wide range of chemical space as 87.5% of elements in the periodic table (91 elements out of 104 elements) were included in BP dataset, 74% (77 out of 104) in log *S* dataset, 98% (102 out of 104) in MP dataset, and 72% (75 out of 104) in PP dataset. The electronic configuration of elements was used as a descriptor for inorganic compounds. Hyperparameters of the models were determined after cross-validation (CV) based grid search over all combinations of them such as neural network architecture, activation function, optimizer, regularization parameters, and dropout ratio. Model performance was measured using *R* square (*R*^2^), mean absolute error (MAE), and Spearman's rank correlation coefficient (SpeaR). BP prediction model was developed and validated with 537 inorganic compounds whose BP ranged from −268.928 to 5590 °C (*R*^2^: 0.88, SpeaR: 0.94, MAE: 222.65 °C on test set). log *S* prediction model was developed and validated with 1008 inorganic compounds whose log *S* ranged from −12.95 to 1.75 (*R*^2^: 0.63, SpeaR: 0.83, MAE: 1.26 on test set). MP prediction model was developed and validated with 1647 inorganic compounds whose MP ranged from −259.16 to 3880 °C (*R*^2^: 0.89, SpeaR: 0.93, MAE: 170.39 °C on test set). PP prediction model was developed and validated with 442 inorganic compounds whose PP ranged from −185 to 1980 °C (*R*^2^: 0.66, SpeaR: 0.76, MAE: 147.55 °C on test set). Developed models achieved sound prediction accuracy for precise prediction of continuous endpoint and prioritization of inorganic compounds.

## 2. Results and discussion

### 2.1. Visualization of structure and property diversity

**Fig. 1:**
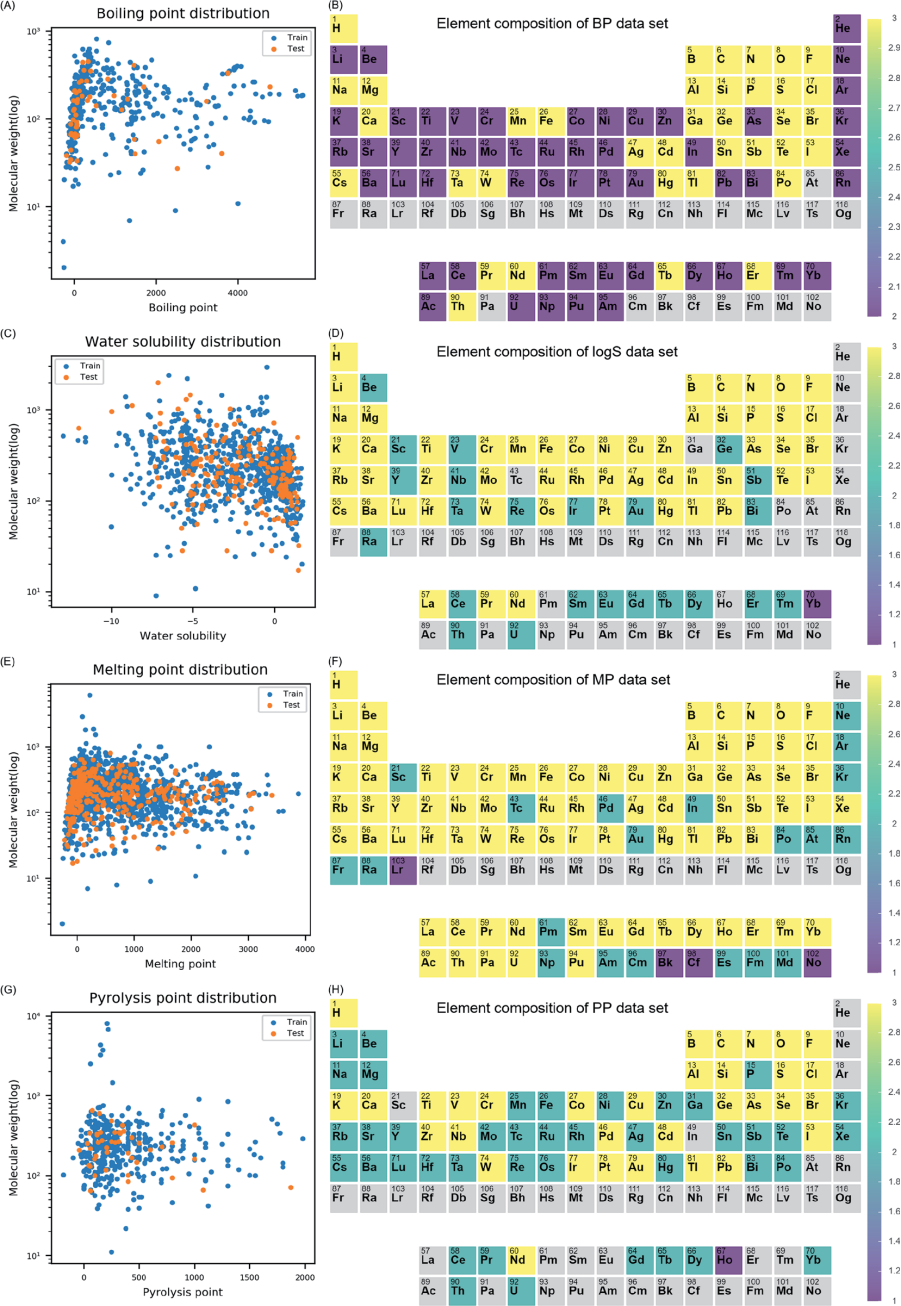
Data distribution in training set and test set were presented with molecular weight and the target endpoint (A, C, E and G). In the periodic table, element composition in training set and test set were presented (B, D, F and H). Color for each element was determined based on group which is marked as three if elements found both training set and test set, two if elements found in training set alone, and one if elements found in test set alone. Color bar next to the periodic table indicates color for each group.

Before model development, data distribution, and chemical space of four datasets (*i.e.*, BP, log *S*, MP, and PP) were examined. The distribution of molecular weight (MW) and each endpoint was visualized for training and test set to understand the diversity of the properties in each data set (Fig. 1). The models were intended to cover diverse inorganic compounds in their applicability domain in terms of element diversity consisting of the compounds. To understand compositional diversity in four datasets, the presence of element among inorganic compounds was visualized on the periodic table (Fig. 1). In the figure, the number of each element according to the molecular formula of the compounds were counted in training and test set. Elements marked with three, two, and one if they belong to both sets, training set alone, and test set alone, respectively. In this study, elements from atomic number 1 (hydrogen) to 104 (rutherfordium) were covered in descriptor calculation. In Fig. 1B, 87.5% of the elements (91/104) on the periodic table were included in the BP dataset. Missing elements within the BP dataset were elements in 7^th^ period, few actinides, and astatine. In Fig. 1D, 74% of the elements (77/104) on the periodic table were included in the log *S* dataset. Missing elements within the log *S* dataset were the noble gases, elements in the 7^th^ period except radium, some of the lanthanides and actinides, and other elements (gallium, technetium, polonium, and astatine). In Fig. 1F, 98% of the elements (102/104) on the periodic table were included in MP dataset. Missing elements were helium and rutherfordium. In Fig. 1H, 72% of the elements (75/104) on the periodic table were included in PP dataset. Missing elements were elements in 7^th^ period, noble gases except krypton and xenon, some of lanthanides and actinides, and other elements (scandium, indium, and astatine). In four datasets, most elements on the periodic table were covered, thus, models developed with the datasets were expected to be effective on inorganic compounds comprised of diverse elements.

### 2.2. Model development

#### 2.2.1. Hyperparameter selection through cross-validation based grid search

Artificial neural network (ANN) models were developed with fully connected (FC) layers, and the introduction of batch normalization (bN) layer right after FC layer brought performance gain in all models. Table 1 summarizes the best set of hyperparameters based on prediction accuracy in CV. In log *S*, MP, and PP models, prediction accuracy of single-layer architecture network was not improved after further hidden layers were added in the model whereas two hidden layer architecture improved prediction performance compared to one hidden layer architecture in BP prediction model alone. The increase in the number of hidden layers of the model results in prediction accuracy improvements toward training set whereas prediction accuracy on external test set was not improved, or even in worst case, deteriorated. The prediction accuracy of ANN model was compared with support vector machine (SVM) and random forest regressor (RFR); however, ANN model outperformed them in all four datasets. According to ANN architecture, all possible interactions between orbital bits in electron configuration descriptors were calculated in the model training process. Since property of a molecule is a result of complex interaction between the electrons, the electron configuration-based neural network performed better than other machine learning methods by modeling interactions between electrons within a molecule. Based on model accuracy, it was concluded that the ANNs efficiently models physical interactions between electrons significant for the target properties through composition alone.

**Table 1:** *n*-Fold cross validation based hyperparameter optimization results^a^

Endpoint	Model	Hyperparameters	*R* ^2^	SpeaR	MAE	MAE/the range	Endpoint range
BP	ANN	hid^1^*: (97, bN), act^2^*: tanh, opt^3^*: Adam, *λ*^4^*: 0.1, dr^5^*: 0.1	0.73 ± 0.13	0.8 ± 0.06	383.28 ± 88.9	6.54%	−268°C to 5590 °C
		**hid: (97, bN, 24, bN), act: relu, opt: RMSprop,** *λ* **: 0.001, dr: 0.03**	**0.8** ± **0.1**	**0.89** ± **0.05**	**322.36** ± **74.68**	5.50%	
		hid: (97, bN, 24, bN, 24, bN), act: relu, opt: RMSprop, *λ*: 0.001, dr: 0.03	0.83 ± 0.05	0.89 ± 0.04	298.93 ± 44.91	5.10%	
	SVM	*γ* ^6^*: auto, *C*^7^*: 3000, *ε*^8^*: 0.03	0.69 ± 0.08	0.81 ± 0.04	405.93 ± 90.53	6.93%	
	RFR	tree^9^*: 3000	0.72 ± 0.15	0.79 ± 0.07	342.66 ± 80.55	5.85%	
log *S*	ANN	**hid: (46, bN), act: tanh, opt: RMSprop,** *λ* **: 0.003, dr: 0.3**	**0.59** ± **0.09**	**0.79** ± **0.04**	**1.38** ± **0.1**	9.39%	−12.95°C to 1.75 °C
		hid: (46, 23), act: tanh, opt: Adam, *λ*: 0.003, dr: 0.1	0.64 ± 0.04	0.79 ± 0.02	1.24 ± 0.07	8.44%	
	SVM	*γ*: auto, *C*: 30, *ε*: 0.3	0.52 ± 0.06	0.74 ± 0.04	1.48 ± 0.13	10.07%	
	RFR	tree: 10 000	0.27 ± 0.05	0.63 ± 0.02	1.7 ± 0.04	11.56%	
MP	ANN	**hid: (105, bN), act: tanh, opt: RMSprop,** *λ* **: 0.1, dr: 0.01**	**0.81** ± **0.09**	**0.92** ± **0.01**	**201.52** ± **21.78**	4.87%	−259.16°C to 3880 °C
		hid: (105, bN, 52, bN), act: sigmoid, opt: Adam, *λ*: 0.01, dr: 0.1	0.87 ± 0.01	0.93 ± 0.00	180.46 ± 13.01	4.36%	
	SVM	*γ*: auto, *C*: 3000, *ε*: 30	0.79 ± 0.01	0.89 ± 0.01	231.19 ± 9.87	5.59%	
	RFR	tree: 10 000	0.66 ± 0.03	0.80 ± 0.02	284.67 ± 13.18	6.88%	
PP	ANN	**hid: (23, bN), act: sigmoid, opt: Adam,** *λ*: **0.1, dr: 0.01**	**0.31** ± **0.18**	**0.55** ± **0.08**	**214.12** ± **35.12**	9.89%	−185°C to 1980 °C
		hid: (93, bN, 46, bN), act: relu, opt: Adam, *λ*: 0.1, dr: 0.3	0.28 ± 0.12	0.58 ± 0.08	209.0 ± 28.38	9.65%	
	SVM	*γ*: scale, *C*: 3000, *ε*: 1	0.23 ± 0.07	0.57 ± 0.1	212.45 ± 19.84	9.81%	
	RFR	tree: 300	−0.34 ± 0.52	0.38 ± 0.1	262.59 ± 34.43	12.13%	

a 1*hid: hidden layer architecture indicating number of hidden nodes in each layer, 2*act: activation function, 3*opt: optimizer, 4*lambda (*λ*): regularization parameter in ANN, 5*dr: drop out ratio, 6*gamma (*γ*): normalization option, 7*C: regularization parameter in SVM, 8*epsilon (*ε*): parameter for no penalty region, 9*tree: number of trees used in random forest model. *Hyperparameters in bold were considered as best performing option for the models.

#### 2.2.2. Performance comparison between composition-based descriptors

In this study, composition-based descriptors were expected to make good prediction accuracy since inorganic compounds in the data sets were already distinguishable by composition alone. Moreover, simplicity of composition-based descriptors makes anyone with basic understanding of chemistry use the model. In order to compare performance of electron configuration vectors from other composition-based descriptors, neural network models were developed with two more descriptors: matminer^25^ and element composition.^15^ Models were developed with the hyperparameters selected from Section 2.2.1 in order to fairly compare the performance difference between descriptors. Table 2 showed that electron configuration-based neural network performed best compared to other two models. Possible reason for lowest accuracy to matminer descriptors is that the features calculated from the matminer is relevant for endpoints significant in novel material design; therefore, they are not well correlated with physicochemical properties. The result that element composition showed better performance than matminer descriptors, implied that neural network performs better with fundamental information since it automatically models relevant relationship between input and output. For that reason, neural network establishes better relationship with electron configuration than with element composition as interactions between electrons underlie interactions between elements.

**Table 2:** Prediction accuracy comparison between different descriptors

Endpoint	Electron configuration				Element composition				Matminer			
	Num.^a^	*R* ^2^	SpeaR	MAE	Num.	*R* ^2^	SpeaR	MAE	Num.	*R* ^2^	SpeaR	MAE
BP	97	0.8 ± 0.1	0.89 ± 0.05	322.36 ± 74.68	91	0.62 ± 0.09	0.83 ± 0.06	411.57 ± 72.31	132	−1.69 ± 3.87	0.24 ± 0.13	876.02 ± 256.19
log *S*	93	0.59 ± 0.09	0.79 ± 0.04	1.38 ± 0.1	77	0.46 ± 0.17	0.76 ± 0.03	1.44 ± 0.12	151	−0.18 ± 0.21	0.19 ± 0.03	2.45 ± 0.12
MP	105	0.81 ± 0.09	0.92 ± 0.01	201.52 ± 21.78	102	0.72 ± 0.07	0.89 ± 0.01	249.50 ± 17.42	126	0.42 ± 0.04	0.52 ± 0.03	458.66 ± 17.38
PP	93	0.31 ± 0.18	0.55 ± 0.08	214.12 ± 35.12	75	−0.53 ± 0.92	0.51 ± 0.11	241.34 ± 41.73	148	0.08 ± 0.08	0.17 ± 0.12	254.23 ± 21.71

a Number of feature used in model development after removing features whose standard deviation was 0.

#### 2.2.3. Prediction accuracy on external test set and prediction error analysis

**Fig. 2:**
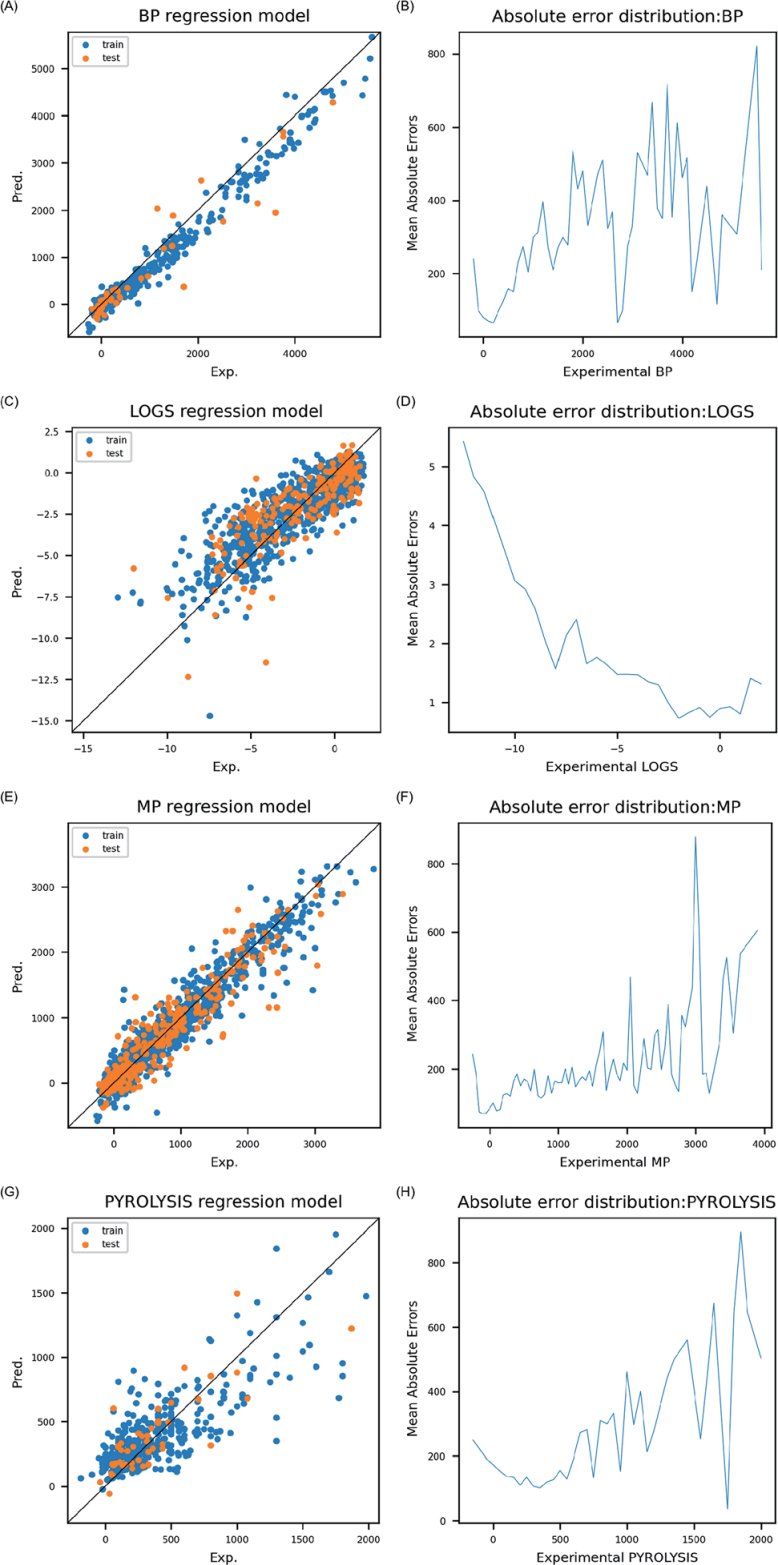
Prediction model accuracy was visualized by comparing prediction result on training set and test set (A, C, E and G). Absolute errors averaged in ranges of the properties were presented (B, D, F and H).

The ANN models were validated with external test sets. Epochs were fixed with 500 in grid search; however, a higher number of epochs could lead to overfitting of the model on the training set, thus, models were tested with different size of epochs, and best prediction accuracies on external test set were presented in Table 3. All models achieved good prediction accuracy as *R*^2^ on test sets were higher than 0.6, and MAE was within 10% compared to entire range of endpoint in the test sets. Particularly, high SpeaR implied that the models were good at ranking inorganic compounds according to each endpoint. Given that major role of molecular descriptors in model building is to differentiate molecules from one another rather than to make precise prediction of the properties, the models in this study can be served as a novel tool for inorganic compounds' descriptor calculation. Particularly, four endpoints studied in this work are significant properties for modeling biologically-relevant endpoints. Therefore, properties predicted by the models are expected to provide valuable information in studying interaction between biological system and inorganic compounds. In comparison between predicted values and experimental values, linear pattern of data distribution was observed in training sets and test sets as four models achieved good robustness and predictability (Fig. 2A, C, E and G). Average absolute error within certain range of endpoint was analyzed by grouping BP data with an interval of 100 °C, log *S* data with 0.5, and MP and PP data with 50 °C. Errors were lower as more data were found within the range (Fig. 2B, D, F and H).

**Table 3:** Performance of the selected artificial neural network models training and external test set

Endpoint	Epoch size	*R* ^2^		SpeaR		MAE		The range of endpoint		MAE/the range	
		Train	Test	Train	Test	Train	Test	Train	Test	Train	Test
BP	500	0.96	0.88	0.95	0.94	178.7	222.65	−268 to 5590	−188.11 to 4785	3.05%	4.48%
log *S*	200	0.73	0.63	0.85	0.83	1.16	1.26	−12.95 to 1.75	−12.00 to 1.49	7.89%	9.16%
MP	500	0.93	0.89	0.95	0.93	136.41	170.39	−259.16 to 3880	−219.67 to 3414	3.30%	4.69%
PP	500	0.61	0.66	0.68	0.76	167.29	147.55	−185 to 1980	−40 to 1870	7.73%	7.73%

#### 2.2.4. Model transferability

In this study, models were developed particularly for inorganic compounds since none of models already developed for small organic molecules, can predict physicochemical properties of inorganic compounds due to significant structural difference between them. In physicochemical property prediction models for organic small molecules, property descriptors or atomic/fragmental compositions were served as input of the prediction models. The structural dissimilarity makes it impossible to calculate already available property descriptors for inorganic compounds, and the compositional difference hinders applying atomic/fragmental composition based models for inorganic compounds. Therefore, model transferability was examined between inorganic compounds alone. Main focus in model transferability is on reliable application of the model to inorganic compounds whose composition was absent in the training set for each model. Since element composition of the compounds in test set is different from that in training set, prediction accuracy on test set was significant in understanding transferability of the models.

In order to check accurate prediction on test set, number of inorganic compounds whose absolute errors were smaller than 10% of entire range of endpoint in the test set, was used as cut-off to count acceptable prediction outcomes within the test sets. 87% of test set (47 out of 54) was within the cut-off in BP, 62% (126 out of 202) in log *S*, 88% (290 out of 330) in MP, and 78% (35 out of 45) in PP. High number of acceptable prediction results in test set supports that the models are transferable to inorganic compounds with diverse composition. Basis of the model transferability was further examined by comparing electron configuration descriptors distribution between training and test set. Even though compositions of inorganic compounds were different, similar electron configuration distribution was observed throughout four data sets (Fig. S1†). This showed that models were trained and validated based on electron composition, and thus weights in the ANN models can be used reliably in prediction of different composition of inorganic compounds. Models developed in this study can possibly be used to predict the properties of organic polymers through transfer learning since composition is also identically significant information to distinguish organic polymers.^26^

#### 2.2.5. Electron interaction analysis

**Fig. 3:**
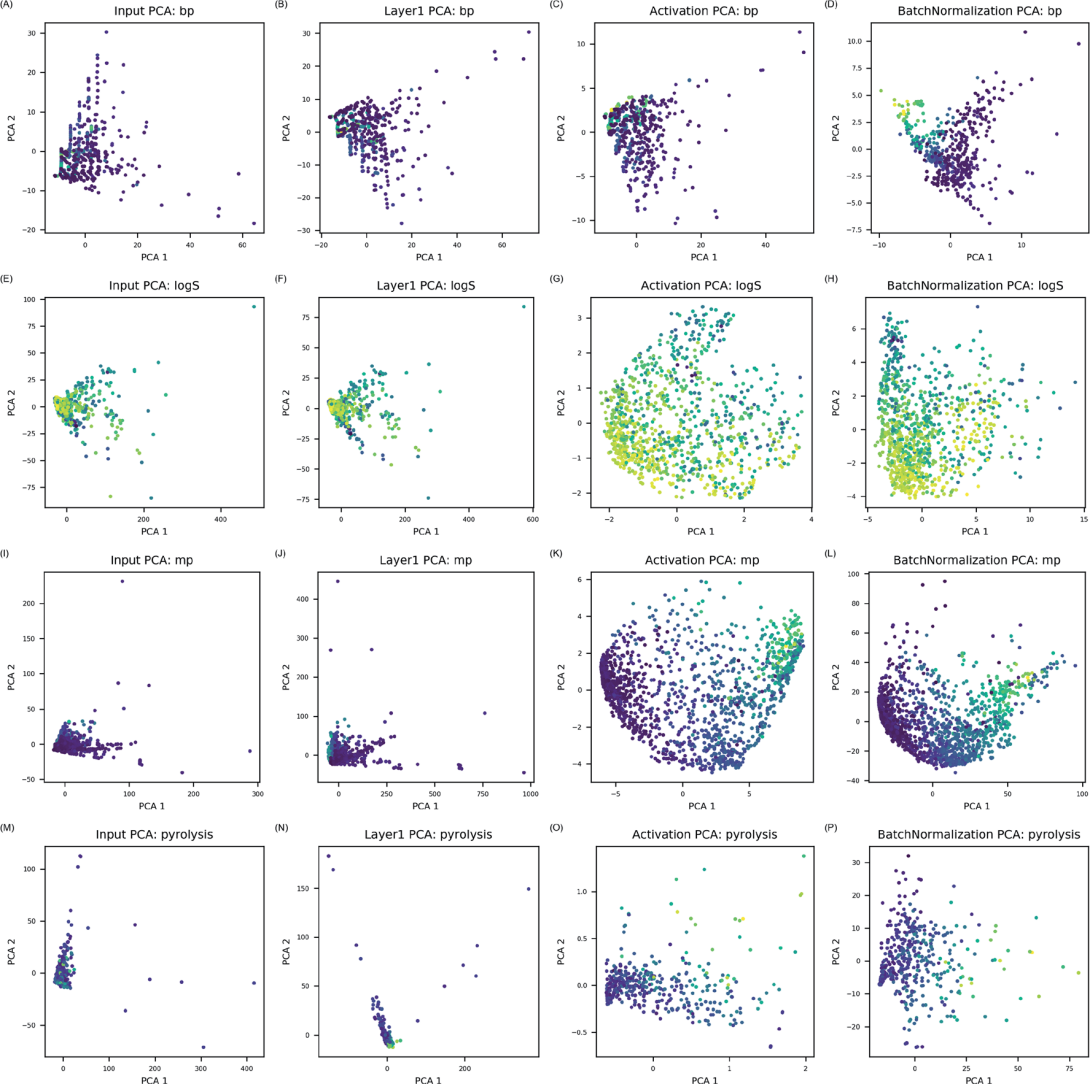
Input PCAs are feature space of electron configuration descriptors (A, E, I and M). Layer 1 PCAs are from linear combination of electron configuration in first hidden layer (B, F, J and N). Activation PCAs are from converted linear combination by each activation function (C, G, K and O). Batch normalization PCAs are from output of batch normalization layer (D, H, L and P). By examining, plots from input PCA to batch normalization PCA, feature space change is visualized layer by layer. Color gradient represents numerical values of the properties. Bright yellow is the highest values whereas dark purple is the lowest.

In order to understand how ANN modeled interactions between electrons, ANN model outputs after first hidden layer, activation layer, and bN layer were compared. Principal component analysis (PCA) was applied to electron configuration descriptors and the outputs for visualization of feature space change in 2D space (Fig. 3). In figure, numerical values of endpoints were shown by color gradient of the points: bright yellow for higher values and dark purple for lower values. In electron configuration level, data points were distributed regardless of the properties. After the descriptor processed through first hidden layer, slight change was observed. From activation layer, data points were spread over the feature space, and data points with high and low values were visually separable except BP data set. After features were processed through bN layer, data points were rearranged according to color gradient of data points. Since color change represents quantitative change of the properties, it is certain that model establishes high correlation between electron configuration and the properties at this states. Feature space change showed that interactions calculated by linear combination of electron configuration descriptors became effective in prediction of the target properties through activation and bN layers.

ANN models in this study modeled complicated interactions between electrons in much simpler way by calculating possible interactions between electrons and transforming them through activation and bN layers. Since electron configuration descriptors in this study considered every atomic orbital identically, activation and batch normalization layers were essential to transform combination of electron configuration descriptors into molecular features relevant for the target property prediction. As transformed features by activation and bN layers identified correlation to the properties, it was concluded that physical interactions between electrons in the compounds were efficiently modeled in data-driven manner for prediction of the target properties.

## 3. Materials and methods

### 3.1. Data collection

Data points for MP, BP, log *S*, and PP were initially collected from CRC handbook of chemistry and physics 97^th^ edition (CRC handbook) in which inorganic compounds and organometallics used in laboratory and industry were selected.^27^ Additional data points for MP and PP were obtained from the work of Igor V. Tetko *et al.*,^28^ and further log *S* data points from AqSolDB,^29^ which were filtered into three groups to select only inorganics and organometallics: (1) compounds without carbon, (2) compounds without hydrogen, and (3) compounds with a metal atom (Fig. S3†). Data points in three groups were then integrated after checking duplicated compounds between the groups. By definition, inorganic compounds are chemicals without carbon–hydrogen bonds; therefore, compounds without carbon (group 1) or hydrogen (group 2) belong to inorganics. To select molecules with a metal atom (group 3), RDKit (http://www.rdkit.org/) was used to filter out molecules containing C, H, O, N, F, P, S, Cl, Br, I, Na, Ca, K, and Si. The list of atoms for filtering was decided since C, H, O, N, F, P, S, Cl, Br, and I are commonly found among molecular structures of pharmaceuticals, and Na, Ca, and K are frequently used as a salt in medication. Properties of organosilicon compounds are similar to organic compounds, hence, compounds with Si were also removed from the set.^30^ In case where compounds were overlapped between CRC handbook and additional data sources with a discrepancy in endpoint, data point from CRC handbook was selected since CRC handbook data were manually curated by experts while additional data sources were curated through automated algorithm, and higher credibility was expected from data manually curated and evaluated by an expert.

BP is normally measured in °C under 760 mmHg pressure, and BP of 538 inorganic compounds were obtained from the CRC handbook. No additional BP data points were obtained for inorganic compounds since all available data for BP was focused on organic compounds (Table S2†).

log *S* is the logarithmic scale of water solubility in mol L^−1^. CRC book reported water solubility in g/100 g H_2_O with the temperature at the point of measurement; therefore, water solubility value was converted into mol L^−1^, and water solubility values measured between 20–30 °C were selected. The water solubility of 459 inorganic compounds were obtained from CRC handbook. Further log *S* data were obtained from AqSolDB in which 9982 log *S* data points were compiled from nine distinctive log *S* database and out of them only 658 compounds were inorganics or organometallics. After integrating two datasets, log *S* of 1008 inorganic compounds were obtained for model development (Table S3†).

MP is normally measured in °C, and MP of 1448 inorganic compounds were obtained from the CRC handbook. MP of inorganic compounds were further collected from the work of Igor V. Tetko *et al.* in which 275 133 MP data points were available and from them only 256 compounds were inorganics or organometallics. Data of 1448 MP from CRC handbook and 256 MP data from the work of Igor V. Tetko *et al.* were combined to increase available data points. After the integration of the two datasets, MP for 1647 inorganic compounds were obtained for model development (Table S4†).

PP is where a compound is decomposed. It is normally reported during experiments for measurement of MP or BP. PP of 439 inorganic compounds were obtained from the CRC handbook. Additional PP data were collected from the work of Igor V. Tetko *et al.* in which 13 769 PP data points were available and out of them only four compounds were inorganics or organometallics. In total, PP of 442 inorganic compounds were obtained for model development (Table S5†).

### 3.2. Electron configuration vector calculation

**Fig. 4:**
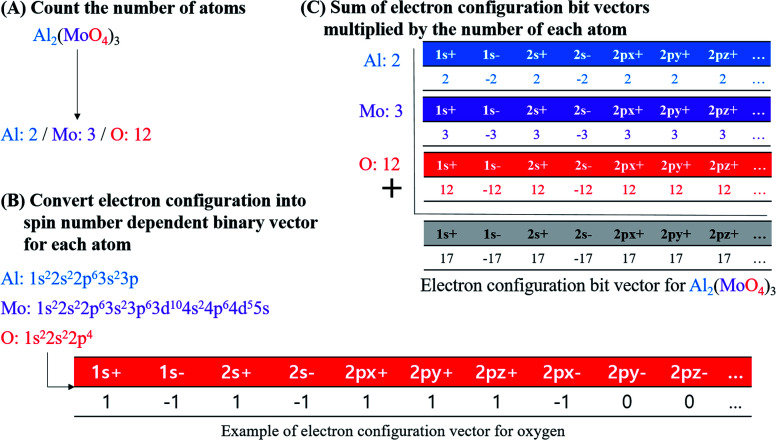
Procedure of calculating the electronic configuration bit vectors for inorganic compounds, taking Al_2_(MoO_4_)_3_ as an example molecule.

In this study, electron configuration vectors for inorganic compounds were calculated as a descriptor for model development. Molecular formula (MF) of inorganic compounds was used to calculate electron configuration vectors (Fig. 4).

First, element types and the number of each element in MF were specified. Element composition table is available in Table S6 for BP dataset, Table S7 for log *S*, Table S8 for MP, and Table S9 for PP.† For example, if Al_2_(MoO_4_)_3_ is an input, element types of the compound are Al, Mo, and O, and the number of each element is two for Al, three for Mo, and 12 for O according to the MF (Fig. 4A).

Second, the electron configuration of each element was obtained (Table S10†).^31^ Ground-state of configuration was assumed for every element consisting of inorganic compounds. In the current implementation, elements from atomic number 1 to 104 were accepted for the descriptor calculation. Atomic orbitals considered in electron configuration vector generation are s orbital for elements in period 1, s and p orbitals in period 2 and 7, s, p, and d orbitals in period 3 and 6, and s, p, d, and f orbitals in period 4 and 5 (*i.e.*, 1s, 2s, 2p, 3s, 3p, 3d, 4s, 4p, 4d, 4f, 5s, 5p, 5d, 5f, 6s, 6p, 6d, 7s, and 7p). Since degenerated p, d, and f orbitals were notated as p_*x*_, p_*y*_, and p_*z*_ for p orbitals, d_*xy*_, d_*yz*_, d_*xz*_, d_*z*^2^_, and d_*x*^2^–*y*^2^_ for d orbitals, and f_*xyz*_, f_*xz*^2^_, f_*yz*^2^_, f_*z*^3^_, f_*z*(*x*^2^–*y*^2^)_, f_*x*(*x*^2^–3*y*^2^)_, and f_*y*(3*x*^2^–*y*^2^)_ for f orbitals, the notation for each orbital were used as indices of electron configuration vector with their period number. Each orbital possesses two electrons with different spin; therefore, the index of electron configuration bits were marked with plus and minus sign, and the bit assigned for each index follows the sign of electron spin. Electron configuration bit vectors are generated according to Hund's rule, empty orbitals are singly occupied first. In singly occupied orbital, a positive sign of bit was assigned first, and then a negative sign of bit was assigned according to the total number of electrons for an inorganic compound. For instance, oxygen's electron configuration is 1s^2^2s^2^2p^4^. Thus, 1s and 2s orbitals are fully occupied whereas 2p orbital is partially occupied with 4 electrons. In case of 1s and 2s orbitals, bit vector contained two plus ones and two minus ones (*i.e.*, [1s^+^]: 1, [1s^−^]: −1, [2s^+^]: 1, and [2s^−^]: −1). For 2p orbital bit vector, [2p_*x*_^+^], [2p_*y*_^+^], and [2p_*z*_^+^] were filled with plus-ones, and [2p_*x*_^−^] with minus one while [2p_*y*_^−^] and [2p_*z*_^−^] with zeros. For the other orbitals, all were filled with zeros as no electrons occupy them (Fig. 4B).

Third, electron configuration bit vector of each atom was multiplied with the number of atoms according to MF, and then each bit in the vectors were added along the indices to produce electron configuration bit vector for an inorganic compound. Electron configuration bit vector for inorganic compounds is in Table S11 for BP dataset, Table S12† for log *S* dataset, Table S13† for MP dataset, and Table S14 for PP dataset.† In the example of Al_2_(MoO_4_)_3_, Al, Mo, and O are present with ratio of 2 : 3 : 12 in the compound. Therefore, Al electron configuration bit vector is multiplied with two, that of Mo with three, and that of O with twelve, *e.g.*, multiplied bit vector for O is as [1s^+^]: 12, [1s^−^]: −12, [2s^+^]: 12, [2s^−^]: −12, [2p_*x*_^+^]: 12, [2p_*y*_^+^]: 12, [2p_*z*_^+^]: 12, [2p_*x*_^−^]: −12, and [other orbital bits]: 0. In summation of multiplied bit vectors for Al, Mo, and O, a bit for the identical orbital indices were added. For instance, a bit for [1s^+^] between Al, Mo, and O was added to produce [1s^+^] bit for Al_2_(MoO_4_)_3_ (Fig. 4C).

### 3.3. Data preprocessing and external test set design

After electron configuration bit vector was prepared for each endpoint, orbital bits whose standard deviation was zero were removed from the dataset since weights for them cannot be properly trained in prediction model owing to the absence of atom type covering the orbital bits. After preprocessing, the length of electron configuration vectors were 97 bits in BP dataset, 93 bits in log *S* dataset, 105 bits in MP dataset, and 93 bits in PP dataset.

Since additional datasets were not available externally, they were divided into training and test sets for the validation of prediction models. As a relatively small number of data was available for BP (538) and PP (442), datasets were divided into training and test sets with ratio of 9 : 1 to use most of data in training the models as MP (1647) and log *S* (1008) data were divided with ratio of 8 : 2 into training and test sets to apply more data for validation. The external test set was prepared by random sampling. For BP and PP data set, stratified random sampling was applied for test set design; however, it gave no difference in randomly sampled test set since entire range of endpoint was large whereas majority of data points were condensed within the short range. Therefore, random sampling was equally applied for all four data sets.

### 3.4. Metrics to measure prediction accuracy

The performance of a model was evaluated with three metrics: *R*^2^, MAE, and SpeaR. *R*^2^ measures the goodness-of-fit of prediction outcomes to evaluate robustness and predictive ability. A model with *R*^2^ higher than 0.6 on the test set is considered as a highly predictive model.^32^ MAE, an average of the absolute difference between experimental values and predicted values, is used to measure quantitative errors in prediction values on average. Even though *R*^2^ is low, a model is considered as useful in predicting the endpoints when MAE of the test set is less than 10% of the whole range of target properties.^32^ MAE and *R*^2^ are calculated as,
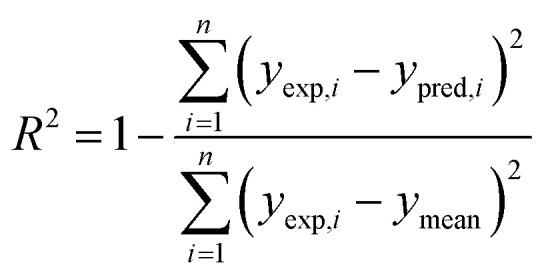

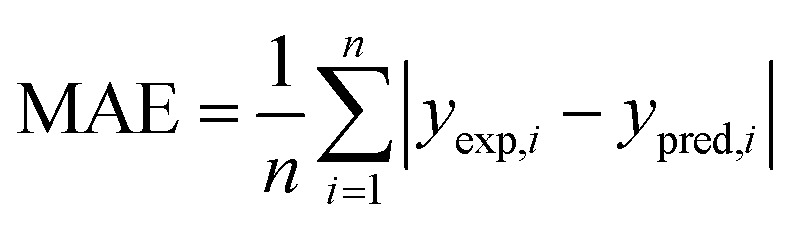
where *n* is the number of data, *y*_exp,*i*_ is experimental value of *i*-th compound, *y*_pred,*i*_ is predicted value of *i*-th compound, and *y*_mean_ is a mean value of the entire *y*_exp_ values.

SpeaR measures how accurately the compounds were ordered by prediction outcome. If compounds were correctly ranked based on predicted values, SpeaR can still be sufficiently high with low *R*^2^ or high MAE.^32^ SpeaR is calculated as,
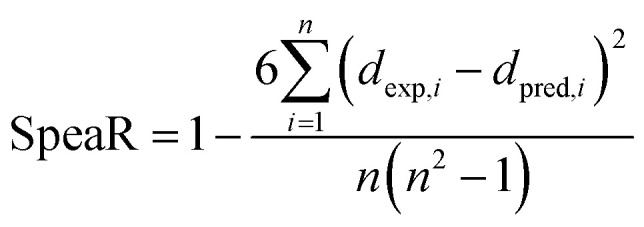
where *n* is the number of data, and *d*_exp,*i*_ and *d*_pred,*i*_ are an order of the compound according to target endpoint and prediction outcomes from one to the total number of compounds. Models with SpeaR > 0.6 were considered as effective since results higher than 0.6 in SpeaR indicate that the model prioritizes data points fairly precisely.^32^

## 4. Conclusions

Even though numerous prediction models already developed to predict the physicochemical properties of small organic compounds, none of them can predict the properties of inorganic compounds due to significant structural dissimilarity. In this study, QSAR models for inorganic compounds were developed to predict BP, log *S*, MP, and PP, which are significant in regulatory perspective. Dataset used in this study not only covered a wide range of endpoints but also diverse elements. The electron configuration descriptors outperformed other composition-based descriptors and showed good prediction accuracy. Molecular properties were determined by complex interaction of electrons, and ANN models efficiently modeled the interactions between electrons significant for prediction of the target endpoints. Accurate prediction outcomes on test set implied that models developed in this study are transferable for prediction of inorganic compounds with novel composition. In ANN model analysis, activation and bN layers played major role in establishing correlation between electron configuration and the properties.

## Conflicts of interest

There are no conflicts to declare.

## Supplementary Material

RA-010-D0RA05873D-s001

RA-010-D0RA05873D-s002
