# Supplementary material for: Electron configuration-based neural network model to predict physicochemical properties of inorganic compounds
Source: RSC Adv. 2020 Sep 8;10(55):33268–78. doi: 10.1039/d0ra05873d (PMC9056678; doi:10.1039/d0ra05873d)
Supplement: RA-010-D0RA05873D-s001 [file RA-010-D0RA05873D-s001.pdf]

Electronic Supplementary Material (ESI) for RSC Advances.
This journal is © The Royal Society of Chemistry 2020

Supplementary

1. Endpoint range and data density analysis

Before model development, data distribution, and chemical space of four datasets (i.e., BP, logS, MP, and PP) were examined. In Figure 1, the distribution of molecular weight (MW) and each endpoint was visualized to understand the diversity of the structure and the properties. In Figure 1A, 537 inorganic compounds in BP dataset were distributed within a range of MW from 2.016 Da to 815.468 Da, and 75% of inorganic compounds (401/537) were found between 50 Da and 300 Da. The total range of BP in the dataset is from -268.928 and to 5590 ℃, whereas 57% of inorganic compounds (304/537) were found to have BP higher than -100 ℃ and lower than 500 ℃. The range of BP from -100 to 500 ℃ corresponds to only 10% of BP range in the entire dataset (600 ℃ /5858.928 ℃); however, 57% of BP data points were condensed within the relatively narrow window of BP. In Figure 1C, 1008 inorganic compounds in logS dataset were distributed within a range of MW from 9.012 Da to 2967.98 Da, and 75% of inorganic compounds (754/1008) were found between 100 Da and 500 Da. The total range of logS in the dataset is from -12.95 to 1.75 whereas 73% of inorganic compounds (736/1008) were found between -5 and 1. The range of logS from -5 to 1 corresponds to 40.81% of the logS range in the entire data (6/14.7), and 73% of the logS data points were found within the range of logS. As the data size of logS is relatively large, the majority of the data are distributed throughout over a wide range of logS. In Figure 1E, 1647 inorganic compounds in MP dataset were distributed within a range of MW from 2.016 Da to 6115.37, and 76% of inorganic compounds (1257/1647) were found between 100 Da and 500 Da. Among entire data points, 85% of inorganic compounds were found between -100 and 2000 ℃ and total range of MP in the dataset is from -259.16 to 3880.0 ℃. The range of MP from -100 to 2000 ℃ corresponds to 51% of the MP range of the entire dataset (2100 ℃/4139.16 ℃); therefore, 76% of data points were broadly distributed within the relatively large window of MP. In Figure 1G, 442 inorganic compounds in the PP dataset were distributed within a range of MW from 11.028 to 1980.0, and 81% of inorganic compounds (356/442) were found between 100 Da and 500 Da. The total range of PP in the dataset is from -185 to 1980 ℃ while 71% of inorganic compounds (312/442) were found to have PP higher than 0 ℃ and lower than 500 ℃. The range of PP from 0 to 500 ℃ corresponds to 23% among PP range of the entire dataset (500 ℃/2165 ℃); however, 71% of PP data points were condensed within the relatively narrow window of PP. Data distribution analysis results led to the conclusion that the external test set should be carefully prepared under the consideration of data distribution since data was not evenly distributed throughout the entire endpoint range.

2. Hyperparameter optimization process

In ANN model development, optimized hyperparameters were the neural network architecture, activation function, optimizer, regularization parameters, and dropout ratio. FC layer was used in ANN architecture with a dropout layer. Each hidden layer was designed with and without bN layers to evaluate performance improvement through batch normalization. Since possible ANN architecture is limitless by changing a number of hidden layers with a number of hidden nodes in it, ANN with only one hidden layer was constructed first to test all possible cases. Based on the result with one hidden layer architecture, the hyperparameters in the two hidden layer architecture were considered. If the performance was significantly improved by increasing hidden layers, the hidden layer was further increased one by one. The number of hidden nodes for each hidden layer was chosen based on simple rules: 1) the number of hidden nodes never exceeds the number of nodes in the following layer, 2) the number of hidden nodes are decreased by a factor of two, and 3) the number of hidden nodes in the last hidden layer are always larger than ten. For instance, the MP dataset initially contained 105 electron configuration bits; therefore, hidden nodes in the first hidden layer can be 105, 52, 26, or 13. In case where the second hidden layer was added, if the first hidden layer had 52 hidden nodes, then the subsequent hidden layer had 52, 26 or 13 hidden nodes. In the grid search of the ANN architecture, the models were developed with increased number of hidden layers one by one until no significant improvement was observed in cross-validation results. Since the increase of a number of weights in the model may lead to overfitting of the model, models with lower number of weights were preferred if the increase of hidden layers or hidden nodes did not present significant improvement in prediction accuracy.

In the grid search, three activation functions were used: sigmoid, hyperbolic tangent (tanh), and relu. Two optimizers were applied: RMSprop and Adam. L2 regularization was applied with a range of regularization parameter such as 0.001, 0.003, 0.01, 0.03, 0.1, 0.3, 1, and 3. A dropout layer was added with a range of dropout ratios such as 0.01, 0.03, 0.1, and 0.3. Each combination was evaluated through n-fold cross-validation to evaluate which hyperparameter worked best for each dataset. 10-fold cross-validation was used for BP and PP, and 5-fold cross-validation was used for MP and logS to make the size of the validation set similar to the test set.

The hyperparameter grid space grows as hidden layer increases; therefore, the range of regularization and dropout was reduced according to the result in one hidden layer model. Hyperparameters that were not applied within the top five model outcomes based on MAE, R2, and SpeaR, were removed from the hyperparameter grid space. To compare the performance of ANN model, SVM, and RFR models were also developed. The SVM model was developed with radial basis function as a kernel. In grid search for SVM, parameter for regularization (C) and epsilon were tested from a range of values such as 0.001,

0.003, 0.01, 0.03, 0.1, 0.3, 1, 3, 10, 30, 100, 300, 1000, and 3000. Gamma was selected from two options: scale and auto. RF models were developed with different numbers of trees such as 10, 30, 100, 300, 1000, 3000, and 10000. Hyperparameter search was performed on NEURON (https://www.ksc.re.kr/eng/resource/neuron) in Korea National supercomputing center. Tensorflow version 2.0.0 was used for ANN model development and scikit-learn version 0.20.1 was used for SVM and RFR model development.

3. Figures

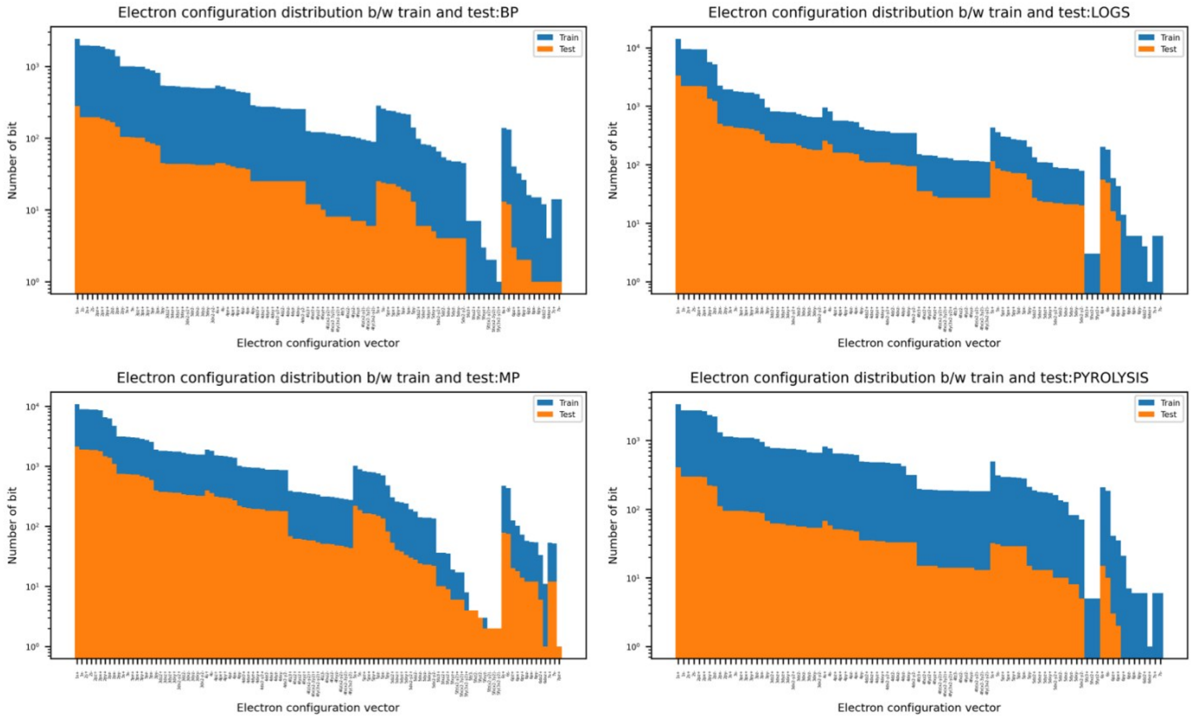

Figure S1. Distribution of electron configuration vectors were presented.

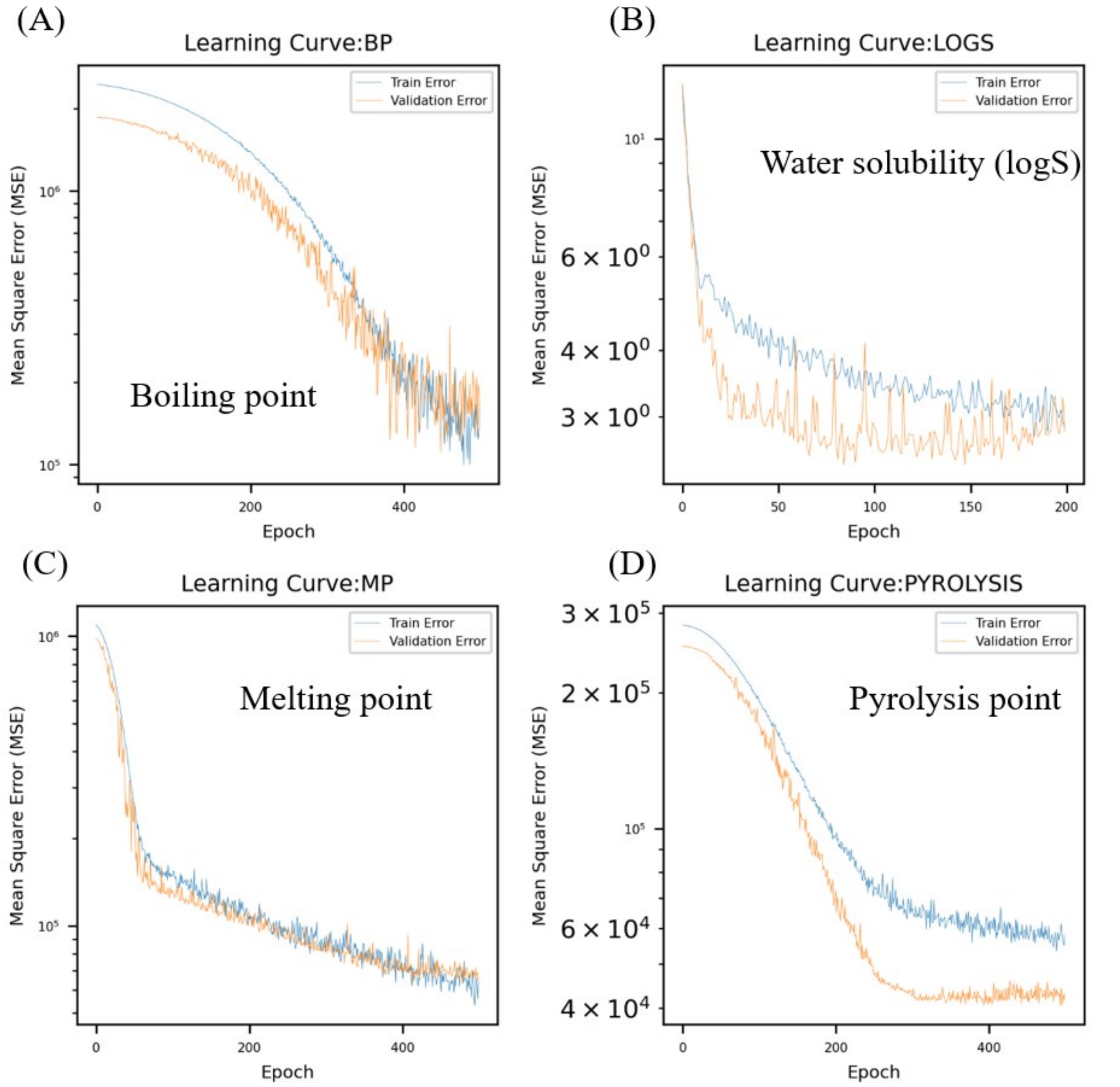

Figure S2. Learning curves of the ANN models: normal boiling point (A), water solubility (B), normal melting point (C), and pyrolysis point (D).

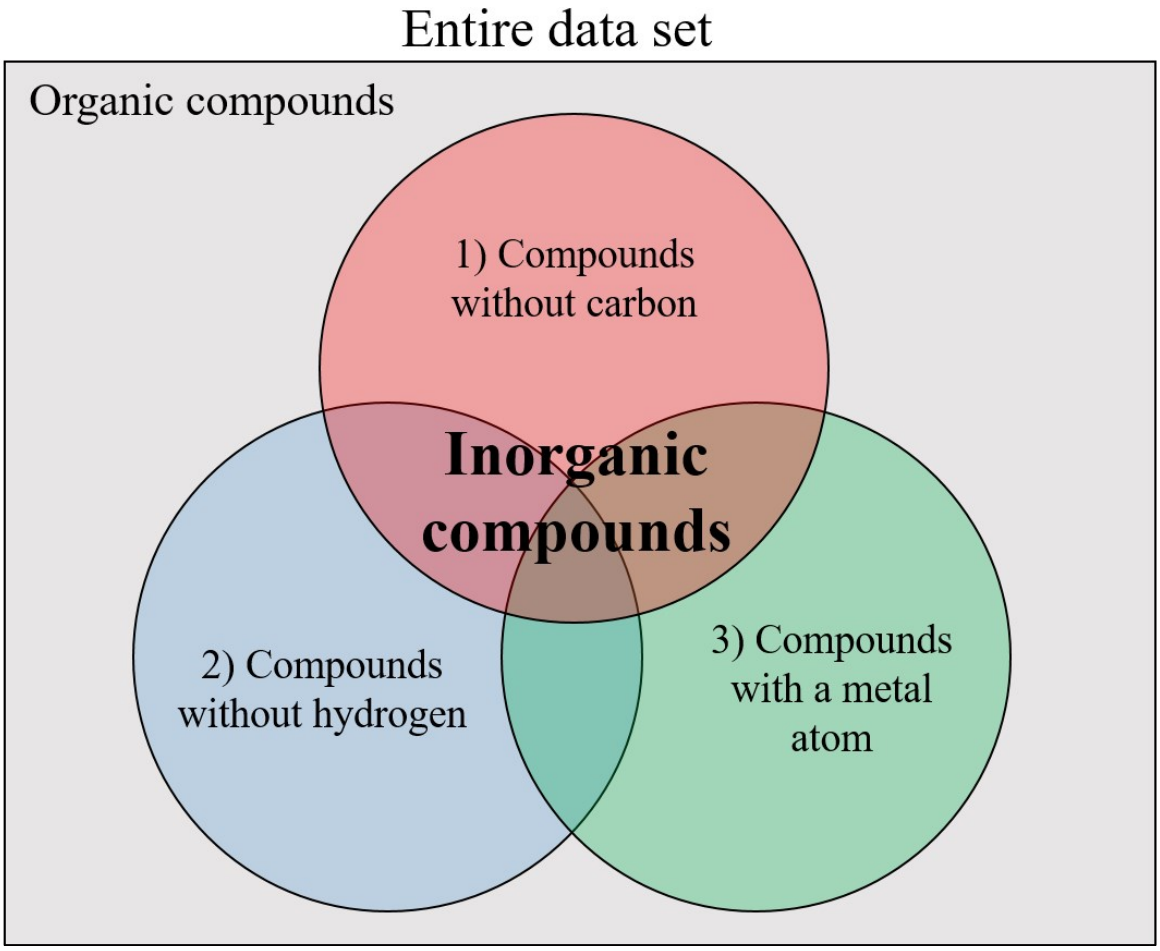

Figure S3. To select only inorganics and organometallics, the data points are filtered into three groups: compounds without carbon, compounds without hydrogen, and compounds with a metal atom.
